# Engagement and Action for Health: The Contribution of Leaders’ Collaborative Skills to Partnership Success

**DOI:** 10.3390/ijerph6010361

**Published:** 2008-01-21

**Authors:** Walid El Ansari, Reza Oskrochi, Ceri Phillips

**Affiliations:** 1 Faculty of Sport, Health & Social Care, University of Gloucestershire, Gloucester, United Kingdom; 2 School of Technology, Department of Mathematical Sciences, Oxford Brookes University, Wheatley, Oxford, United Kingdom; E-mail: roskrochi@brookes.ac.uk; 3 Institute for Health Research, School of Health Science, Swansea University, Singleton Park, Swansea, Wales, United Kingdom; E-mail: c.j.phillips@swansea.ac.uk

**Keywords:** Partnership, coalition, community-based, inter-professional, multi-site evaluation, health professions education, leadership

## Abstract

A multi-site evaluation (survey) of five Kellogg-funded Community Partnerships (CPs) in South Africa was undertaken to explore the relationship between leadership skills and a range of 30 operational, functional and organisational factors deemed critical to successful CPs. The CPs were collaborative academic-health service-community efforts aimed at health professions education reforms. The level of agreement to eleven dichotomous (‘Yes/No’) leadership skills items was used to compute two measures of members’ appreciation of their CPs’ leadership. The associations between these measures and 30 CPs factors were explored, and the partnership factors that leadership skills explained were assessed after controlling. Respondents who perceived the leadership of their CPs favourably had more positive ratings across 30 other partnership factors than those who rated leadership skills less favourably, and were more likely to report a positive cost/ benefit ratio. In addition, respondents who viewed their CPs’ leadership positively also rated the operational understanding, the communication mechanisms, as well as the rules and procedures of the CPs more favourably. Leadership skills explained between 20% and 7% of the variance of 10 partnership factors. The influence of leaders’ skills in effective health-focussed partnerships is much broader than previously conceptualised.

## 1. Introduction

The objective of this paper is to further the understanding of the links between leadership skills and a wide range of process and outcome factors of effective partnerships in public health. Collaboration across professional and agency boundaries has taken many approaches, involving shared decision-making, pooled budgets and integrated provision [1–6]. Partnerships are becoming the norm for capacity building and development in public health, health education and disease prevention [7–11]. Many granting Foundations such as W.K. Kellogg [12] have supported local partnerships for community-wide planning to achieve health objectives.

We use the terms ‘coalition’ and ‘partnership’ interchangeably to indicate the process by which stakeholders invest themselves with ideas, experiences and skills that collectively bear upon problems through joint decision-making and action [13]. Community partnerships (CPs) of professionals and community-based grassroots agencies influence long-term health and welfare, nurture social inclusion, reduce inequalities, and instil a sense of community [14]. However, these community-wide initiatives require extra effort and time where professionals and ‘lay’ people collaborate on an equal power basis, with respected inputs and similarly heard expressions [15]. Such partnerships face multiple internal and external ‘dynamic tensions’ [16, 17], and require effective leadership [18, 19].

Leadership is a ‘coalition-building’ factor associated with implementation, maintenance, organisation and effectiveness [20–22]. Leaders advance equal status, encourage joint working and enhance partners’ involvement in decision-making. Such actions increase members’ participation, satisfaction and commitment [23, 24]. Leaders’ characteristics, personal features and decision-making styles influence positive team outcomes [25], while leadership qualities, knowledge, commitment, competence, communication and interpersonal relations are critical in realising objectives [26]. In empowering agencies, leaders promote members’ cohesion and involvement in planning [27, 28].

In addition, successful CPs build on other factors: broad stakeholder and community representation, administrative/ management skills and quality communication [7, 29–31]; and staff and lay members’ expertise and experience [11, 32, 33]. The costs and benefits of participation are important [34, 35], as they enhance member and resource allocation satisfaction [36]. A supportive organizational climate with clear rules, procedures and roles promotes operational understanding in CPs [37], while collaborative decision-making and positive interactions build equitable staff-constituency relationships [30] that endorse a sense of ownership and community [37, 38].

This study was informed by multiple conceptual frameworks for successful CPs. These included membership, organisational and structure characteristics, resources and support, and function and roles [39–41]. The frameworks also addressed operational parameters (leadership and management skills, communication, decision making processes) [27, 28, 42–44] that require democratic, visible and supportive leaders [41].

Four aspects emerged from the literature: 1) CPs are vital in tackling common health concerns across partners and communities; 2) effective leadership is essential for CPs to achieve their health outcomes; 3) leadership does not exist in isolation and many non-leadership factors neatly interlace in effective CPs; 4) leadership is related to team efficacy, satisfaction, and outcomes [41], but current models fail to fully explain relationships between leadership and the factors that contribute to successful CPs.

Few have systematically examined the relationships between leadership and partnership outcomes [45]. Kumpfer *et al*. [41] emphasised the lack of understanding of the influence of leadership, proposing a model where leadership was related to team efficacy, satisfaction, and outcomes. This paper builds on Kumpfer’s model to further the understanding of leadership’s influence. It hypothesises that leadership will not only influence team efficacy, satisfaction, and outcomes, but also many other factors critical to successful CPs. The term ‘factor’ describes any given feature of effective CPs (e.g. flow of information, communication, interaction, or commitment factors), and ‘item’ indicates the number of questions that comprised each factor.

### 1.1. Aims of the Study

We explored relationships between CPs’ leadership qualities and many operational, functional and organisational partnership factors. The analysis is part of a wider survey into 5 CPs in South Africa [8]. The objectives were to:

- describe the CPs’ aims and outcomes
- compute each participant’s level of agreement on 11 dichotomous (‘Yes/ No’) questionnaire items relating to leadership skills in their CPs; employ this to generate two related measures: a continuous Leadership Skills Score (LSS) and a Leadership Skills Category (LSC); and, explore the relationship between them. [LSS was employed to explore the association between leadership skills and CP factors measured with 2 or 3 categories; LSC was employed to explore the association between leadership skills and CP factors with continuous scales and categorical scales measured with more than 4 categories].
- test the assumption that the LSC would differentiate among participants’ levels of engagement and involvement in the CPs by employing 10 confirmatory items to confirm the predicted direction of the results prior to the main analyses
- assess whether members with greater level of LSC would also experience more positive perceptions of 26 partnership factors, as well as a greater benefit-cost ratio for their participation
- assess whether participants with greater LSS would also experience more positive perceptions of another three partnership factors (operational understanding, communication mechanisms, and rules and procedures)
- explore the partnership factors that leadership skills contribute to explaining across the participating CPs and their implications; and
- use the findings to revisit Kumpfer *et al*.’s [41] model where leadership was a major factor related to team efficacy, satisfaction, and outcomes.

### 1.2. Background: The South African CPs for Health Professions Education (HPE)

Access to public services in South Africa was skewed [46], and a policy aim was to increase the number of students who enter primary care and work with disadvantaged communities. Kellogg Foundation facilitated this by establishing CPs of tripartite academic-health service-community stakeholders who leveraged institutional change from outside through partnering with the communities for more primary care practitioners. Similar efforts elsewhere were effective [47].

The CPs were delivering many outcomes across all stakeholders. For example, HPE outcomes included knowledge acquisition and socialisation, premised on what students experienced, and the setting in which this happened [48], while curricula were redesigned to be more community-based, and linked with community resources/settings that enabled students’ participation [13, 49, 50]. Service outcomes focussed on multi-professional teams for community-responsive primary care [51]. Health professions student outcomes comprised educational shifts to ‘generalist’ training that prepared community-appreciative providers [52, 53]. Community outcomes included active lay involvement in HPE, specific roles in the educational process [19] and better understanding of the university [54, 55]. Policy outcomes were the collective impact of CPs on HPE policy change away from traditional clinical training [56]. Sustainability outcomes included partner involvement, role clarity, relationships and group ownership [5, 40] for long-term viability with the reallocation of resources [57]. Finally, structural change outcomes were service delivery reforms and community linkages to facilitate the changes for lay and professional agencies involved in the efforts [55, 58].

## 2. Methods

### 2.1. Sample and Tools

Participants (N = 668) were members of five CPs, each serving populations ranging between 35,000 and 300,000. The study tool was a self-administered questionnaire compiled from surveys of health coalitions and Kellogg evaluation tools [27, 39, 40, 59 – 61]. Some items were slightly modified to fit the objectives of the CPs under study. The instrument is detailed elsewhere [35]. Box (1) depicts 11 items that comprised the leadership skills factor, to include leaderships’ incentives, styles, actions and management [27].

**Box 1. Leadership skills: eleven items.**

**The Partnership leadership**
1. Provides me with a lot of good information
2. Reports our accomplishments through newsletters, etc.
3. Makes me feel welcome at meetings
4. Gives praise/ recognition at meetings
5. Solicits my opinions and comments during meetings
6. Asks me to assist with organizational tasks
7. Intentionally seeks out and welcomes my views
8. Intentionally seeks out the views of other people outside the Partnership
9. Provides me with continuing education opportunities
10. Holds social gatherings for Partnership members
11. Offers group activities (tours of other Partnerships, etc.) to Partnership members

All are categorical dichotomous items, scored on (‘Yes/No’) format; two items inquired about information provision/reporting of achievements; three items inquired about consultation, recognition and solicitation of opinions; three items were about involvement skills and welcoming of views of those within/outside the CP; three items queried leaderships’ promotion of continuing education, social gatherings and group activities.

Table 1 comprises description, number of items and internal consistency of 30 factors that addressed CPs’ characteristics, processes, structures and outcomes thus reflecting the breadth of this inquiry.

In addition to leadership skills (1 factor) and another 30 partnership factors, 10 further items of engagement and involvement were included as confirmatory items (e.g. time since joining the CP; percentage of CP meetings attended; time spent on CP activity; number of times stakeholders recruited new members, served as CP’s representatives, implemented CP-sponsored events, worked on CP committees, or held CP committee or team leadership positions). As a safety check prior to the main analyses, these were employed in the initial analysis to confirm that LSC differentiates among participants in the predicted direction.

### 2.2. Statistical Analysis

The Statistical Package (SPSS v14) was used to generate two indicators that captured how members gauged the skills of their CPs’ leadership.

- *Leadership Skills Score* (LSS): a quantitative score for each respondent premised on percentage of ‘Yes’ answers to 11 leadership items. LSS ranged from 0–1, where the closer it was to 1 (if all responses were ‘Yes’), the higher was the respondent’s assessment of their CP’s leadership (assuming all items are equal in weight). LSS was then employed to explore the association between leadership skills and 3 CP factors measured with few (2 or 3) categories (see Table 5).
- *Leadership Skills Category* (LSC): LSS was used to generate a measure with 4 categories of leadership skills: ‘Low’ LSC (≤3 positive ratings); ‘Moderate’ (4–6 positive ratings); ‘High’ (between 7–8); and ‘Excellent’ LSC (>8 positive ratings). LSC was employed to explore the association between leadership skills and CP factors with continuous (Tables 2 & 3) and categorical scales (Table 4).

Cronbach’s α indicated the internal consistency for multi-item factors. Pearson correlation matrix assessed correlations between factors. Independent sample t-tests or Analysis of Variance (ANOVA) explored associations between LSC and CP factors measured with continuous scale (Table 3) or between LSS and CP factors measured with few categorical scales (Table 5). Chi-square (χ^2^) tests explored associations between the LSC and CP factors measured with categorical scales (Table 4). Significance level was *P* <0.05. Regression analysis was undertaken in order to explore the contribution of leadership skills to the range of partnership factors under investigation.

## 3. Results

### 3.1. Response Rates and Reliability

The denominators required for response rates were difficult to ascertain. Some ‘potential’ respondents had not attended any CP meetings (inclusion criterion). Others (academicians on CPs’ Boards) apologized that they were not wholly involved. Core Staff (CPs’ paid employees) numbers were verifiable and their response rate was ≈ 90%. For academics/ health services, usually representatives of given units actively participated in the CPs, so ‘snowballing’ helped to reach, follow up and survey eligible members (response rate >90%). The questionnaire’s multi-item scales had excellent/ very good reliability, where for >80% of the scales, α was ≥ 0.70 (range 0.93–0.66, very few sections between 0.70–0.66). All items within each factor contributed positively to internal consistency in all multi-item scales and were retained (Table 1).

### 3.2. Demographic Characteristics of Sample

The sample (*N*=668) comprised community constituencies (*n* = 367) of civic organisations or attending on their own behalf; academic institutions (*n* = 130); health services (*n* = 111); and core staff (*n* = 60). Membership ‘size’ differences existed across CPs and stakeholders. Members’ mean age was 40 years (range 18–78), with differences across CPs (p<0.002), where 90% of sample was >25 years old. There were more females (M= 64%), with variations across CPs (42% – 79% of membership, p<0.001). Overall 78% of respondents reported ‘Black’ ethnicity, which varied (40% – 98%, p<0.001) by the location of CPs within South Africa.

Few (11%) members had previous experience of partnership working (Mean = 3.5 years), but the number of these individuals varied across sites (range 4% – 16% of respondents, p<0.004). Members joined their CPs since ≈ 22 months, but duration varied (range 18 – 27, p<0.001) due to differences in the periods that community (p<0.001) and health services members (p=0.04) had been involved. Academics showed the earliest involvement (M=27.4 months), followed by core staff (M=22 months), and community and health services (M=21 months for both).

Across sites, 45% – 76% of respondents reported ‘Moderate’ or ‘Low’ involvement in their CPs (p<0.001). Participants attended about half CPs’ meetings that they were expected to attend, and attendance varied across CPs (range 38% – 71%, p<0.001). There was disparity in the number of hours per month that members spent on CP work (range 11.7 – 53 hours, p<0.001). Finally, 27% of the sample reported their authority to make decision on behalf the agencies they represented at CP meetings, but with variations across sites (range 14% – 45%, p<0.001).

### 3.3. Partnership Factors

Table 1 depicts the description and reliability of the partnership factors. Members felt the management capabilities in their CPs’ to be above average, but the CPs needed broader representation of local stakeholders. They perceived favourably the staff-community communication, while communication between community members was good, with useful information exchange.

Although partnership work is often voluntary respondents reported above average level of benefits from participation. General satisfaction with their CPs was also average, but satisfaction with resource allocation was lower. Members valued the expertise that health services and academics brought to the CPs slightly more than the skills of civic and community members. They felt that their CPs’ engagement in policy activities could be improved, although they positively rated the partners’ involvement/ effectiveness in some policy areas. However, there were higher levels of engagement in HPE than in policy activities, and good partner involvement/ effectiveness in educational efforts.

Stakeholders had sense of ownership and fair commitment to the efforts. Interactions and consultative decision-making were above average, accompanied by consensus that CPs would achieve their intended outcomes. Generally, members did not feel their material, time and effort contributions to the CPs as excessive, and reported them to be at modest affordable costs. Members perceived the organizational/personnel barriers as minor problems, but felt that CPs’ effectiveness could be enhanced, and rated their individual CPs’ activity in the prior 2 years as moderate. There was role clarity in the inputs that partner agencies typically had in advice on and development of CPs’ operations, and above average operational understanding of CPs’ committees, mission and structure. Members felt that CPs’ communication mechanisms could be enhanced, although a reasonable number of members knew their CPs’ rules/ procedures.

### 3.4. Confirmatory Items of Engagement and Involvement

Across 10 confirmatory items, higher LSC was associated with mainly positive perceptions to the items (except for the first item, see Table 2 below). Members with higher LSC had joined their CPs for longer periods than those with lower LSC, attended more meetings, and spent more time on CP activity. Since joining their CPs, they recruited more new members to the CPs, served more times as CP’s representatives and on more CP-sponsored events, worked on more CP committees and held more leadership positions. In many instances there was an ascending pattern in the ratings of engagement/ involvement items as one moved from lower to higher LSC. These findings confirmed LSC as a valid indicator that distinguished, in the predicted direction, among members with various involvement levels. Interestingly, more members with past CP experience were associated with low/ moderate LSC. This suggested that past experience in partnership settings could cause members to be more critical in their assessment of leadership skills in their current CPs. However, the differences were not significant (Table 2, first row)

### 3.5. Leadership Skills and 26 Partnership Factors

Respondents who rated LSC in their CPs as ‘High’ or ‘Excellent’ consistently scored better on 26 other different partnership factors than those who reported ‘Low’ or ‘Moderate’ LSC (Table 3). In most cases, there was more positive perception across the 26 CP factors as one moved from ‘Low’ leadership skills to those who felt ‘Excellent’ LSC. Positive feelings about leadership were consistently accompanied by partners’ positive perceptions of other CP factors.

### 3.6. Leadership Skills and Members’ Costs/ Benefits Ratio

Table 4 shows that members who reported ‘Low’-’Moderate’ LSC were more likely to feel that their participation entailed more difficulties and costs than benefits. Conversely, those who reported ‘High’-’Excellent’ LSC felt that their involvement had more benefits than difficulties.

As regards day-to-day operations, Table 5 depicts that respondents who rated positively the operational understanding, communication mechanisms and rules and procedures of CPs exhibited, generally, a higher LSS than those who rated these less favourably. Positive perceptions about the CPs’ leadership (higher LSS) were associated with more positive perceptions about CPs’ procedures/ operations, and that communication mechanisms between partners and stakeholders were good and varied.

### 3.7. What Critical Partnership Factors do Leadership Skills Contribute to?

**Figure 1.**
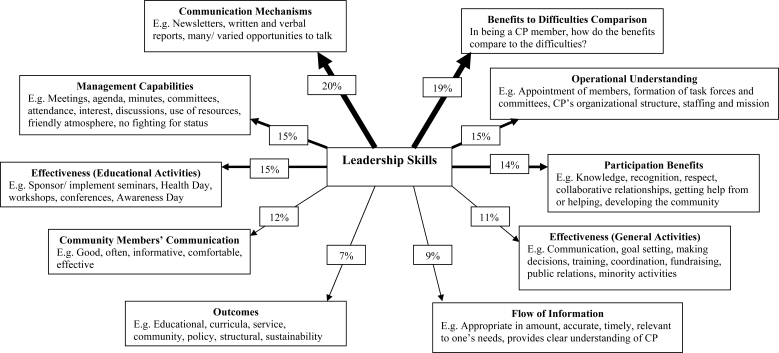
Leadership Skill’s contribution to some Critical Factors across Five Partnerships*. * Standardised effect when controlling for all other factors, thicker lines indicate that leadership skills explained more of the factor/s across the five community partnerships; CP: community partnership.

Leadership Skills contributed explanatory power to the variance of 10 CP factors after controlling for all the partnership factors (Figure 1). It explained 20% and 19% of the variance of communication mechanisms and respondents’ perceptions of the benefits to difficulties of being a CP member; and contributed 15% to another three factors (management capabilities, operational understanding, and effectiveness of the CPs’ educational activities). It also explained the variance of participation benefits (14%), community members’ communication (12%), effectiveness of the CPs’ general activities (11%), flow of information (9%), and outcomes of the partnerships (7%). Favorable perceptions of the partnership leaders by the members were critical to a range of factors of effective partnerships.

## 4. Discussion

In CPs, local political, business, grassroots and civic leaders unite around a community agenda to develop coordinated responses to community health and social challenges [62]. Successful partnering requires effective leadership [63], and leadership style was consistently associated with effectiveness [20]. The CPs influenced health practitioners to be more community sensitive, so partnership leaders need an understanding of the health care system, providers, universities and communities to develop strategies to influence the health system [64]. Leadership is critical in coalitions [65], where leaders relate to their environment, building teams or collaborations [66, 67]. Besides authority, power and influence to guide members to goals [68] leaders require training and technical assistance to promote coalition building/sustainability [69, 70].

In this cross-site evaluation of 5 CPs [71] the tool addressed operational and organisational ‘process’ and ‘outcome’ factors, balancing process measures of how coalitions work and outcome indicators of whether CPs make a difference [72]. Process measures show how close coalitions are connected to the grassroots [38], and are essential to assess effectiveness [5, 73].

For some stakeholders, response rates were challenging to compute. This was not unusual: frequently in collective action, only a fraction of people/organisations with shared interests become involved [74, 75], usually at the minor level of belonging to an agency and paying dues.

The survey tool displayed excellent internal consistency, essential in evaluations of partnerships [73] (α>0.60 is acceptable, but values >0.80 are preferred). In this study, >80% of scales of multi-item measures had α ≥ 0.70 (range 0.66 – 0.93), supporting values reported by others [37, 40, 41, 76]. However leadership is often measured in different ways: incentive management [39], task focused [77], shared leadership [30], empowering or collective [36], or embracing multiple features [78]. The 11 features of leadership employed in the present analysis catered for a variety of leadership aspects, behaviours and styles.

In terms of the first objective, the CPs targeted many educational, social, and community development objectives, as with similar efforts elsewhere [10, 47, 65, 79]. The initiative was sharing of models of academic-community partnering collectively focussed on the health needs of the population groups, communities and individuals concerned. Hence, academics had participated slightly longer, reflecting their initial involvement in the ‘pre-formation’ phase [80, 81].

Secondly, it has been shown that it was appropriate to use LSS and LSC when exploring associations between the leadership skills and CP factors. Thirdly, LSC was valid in differentiating among participants across 10 confirmatory items, where higher LSC was associated with positive perceptions to the items. The findings were in the predicted direction, and confirmed the consistency of LSC in differentiating among participants with different engagement levels. An exception was in relation to past CP experience.

Fourthly, affirmative feelings of partners about their leadership were consistently accompanied by positive perceptions across many factors of CPs’ functioning. Others [82] similarly identified 27 measures of coalition characteristics, where many measures were related to leadership performance. Indeed successful university-community collaborations for health curricula reforms require leadership strategies (e.g. consistency, range of leadership behaviours, participative governance) that are associated with positive outcomes [65].

These findings support that member, staff and organisational factors are intertwined in CPs [12, 39–41]. For instance, high LSC partners felt the personnel barriers in their CPs to be less threatening, a critical perspective for a coalition’s internal functioning, where high member turnover, low interest or infighting is disruptive [83]. Further, high LSC participants rated the CPs’ interactions more agreeably, confirming that CPs are flexible/ permeable structures interacting with their environments rather than tightly-bounded entities [84]. Similarly, high LSC members felt more sense of ownership, which promotes greater community participation [38], and valued the staff and community skills, highlighting that member expertise is vital for effective CPs [39, 77, 85].

High LSC members reported an effective information flow and that communication between staff and community members was good - a significant predictor of coalition satisfaction [18, 39]. They reported that their constituencies were more committed, an important factor as CPs’ leaders motivate their members’ commitment, nurturing it into a vision [77, 86, 87]. Different commitment levels result in varied investments of time, effort and resources [28, 88].

Partners with lower LSC felt that their participation entailed more difficulties and costs than benefits than those with higher LSC. Such reciprocity provides insights into whether to participate [35], the benefits/costs of alternative modes of structuring coalitions [84], and the importance of a favourable benefit/cost ratio [27, 89]. Active leaders may accept an equal ratio of benefits to costs [90].

For objective five, higher LSS partners felt positive perceptions on operational understanding of the CPs’ operations, communication mechanisms, and knowledge of CPs’ rules/ procedures. These findings are supported by other studies. Communication is a predictor of intermediary measures of coalitions [39], where open, frequent and varied communication channels are valued [77, 91]. Similarly, knowledge of the CPs’ rules/ procedures is critical, where members’ knowledge of coalition functioning affected later sustainability [92], awareness of rules/ procedures was predictive of agency commitment [39], and both were indicators of CP effectiveness [78, 93].

Regarding the last 2 objectives, leadership skills contributed to explaining the variance of 10 partnership factors ranging between 20% (communication mechanisms) to 7% (outcomes). This represents a ‘net’ effect, after controlling for all the factors under study [94]. The outcomes were provision of primary care services; influencing HPE; and increasing the medical, nursing and other health professions who practice primary care in underserved areas. Revisiting Kumpfer’s [41] model where leadership was related to three factors (team efficacy, satisfaction, and outcomes), the hypothesis of the paper is affirmed: leadership was not only associated with these three factors, and this study extended the associations of leadership to 30 factors, highlighting the importance of leaders’ skills in effective health-focussed partnerships.

## 5. Conclusion and Implications

In CPs public/private agencies, community leaders, academic and health services come together to tackle public health. Voluntary participation between partners who traditionally have not collaborated together requires skilled leadership. Members who perceived favourably the leadership of their CPs consistently scored better on 30 different partnership factors than those who rated leadership skills less favourably. The findings systematically examined relationships between leadership and many partnership processes and outcomes to emphasise the relevance of leadership skills. For researchers, this highlights the importance of including leadership features when undertaking coalition inquires in order to further the understanding of its intricate relationships and pre-requisites as regards stages of coalition formation. For CP practitioners, administrators, directors and coordinators, the findings demonstrate that their inputs, decision-making, interactions, communication and engagement are carefully viewed by partnership constituencies and simultaneously influence CP’s success. For policy makers, this highlights the need for developing and nurturing structures that provide appropriate leadership skills that are supportive and conducive to effective leaders from diverse stakeholders; as well as instilling appropriate incentives for leadership development at different levels. For grant-making bodies, this translates to highlighting the effects of appropriate leadership to potential grantees, encouraging and ensuring the inclusion of leadership technical assistance and training within a partnership’s budget as appropriate, as well as promoting the assessments of leadership aspects in partnership evaluations. Collectively such actions should make a difference.

## Figures and Tables

**Table 1.** Community partnerships: factors, † their description and reliability.

Factor	Description	Number of Items	Mean	Alpha*
Leadership skills	Leaders use incentive management skills	11	0.71	0.78
Management capabilities	Effective management processes and policies	22	4.94	0.93
Community representation in the CP	Perception that CP is representative of the community	1	2.65	—
Staff-community communication	Quality of staff-community member communication	5	4.58	0.91
Community communication	Quality of community member-member communication	5	4.79	0.92
Flow of information	Amount, accuracy, timing, relevance of information	5	4.63	0.68
Participation benefits	Benefits accrued to participant and organisation	11	5.17	0.90
Satisfaction with the CP	Satisfied with CP operations/ accomplishments	5	4.63	0.84
Resource allocation satisfaction	Satisfied with use of CP funds in the community	1	3.84	—
Staff expertise	Abilities as change agents, working with / organising community groups, implementing educational activities, maintaining the CPs	11	5.07	0.91
Community member expertise		11	4.63	0.90
CPs’ engagement in policy activities	Engagement in policy/ advocacy activities	1	4.05	—
CPs’ effectiveness in policy activities	Partners’ involvement/ effectiveness in policy/advocacy activities	2	5.10	0.80
CPs’ engagement in HPE education	Engagement in educational activities	1	5.36	—
CPs’ effectiveness in educational activities	Partners’ involvement/ effectiveness in educational activities	2	5.40	0.82
Sense of ownership	Committed, feels pride, cares about the CP	4	5.31	0.76
Organisational commitment	Endorsed/adopted CPs’ missions; cosponsored efforts	4	5.17	0.79
Interactions within the CP	Interactions, conflict, differences, control among partners	7	4.80	0.81
Decision-making	Attitudes/ beliefs related to participation in the CP	9	4.73	0.67
Outcomes	Confidence that CP will influence HPE/PHC	16	4.72	0.93
Contributions to the CP^a^	Extent to which partners/organizations make contributions	4	3.87	0.72
Participation costs^b^	Participation in the CP is difficult	5	3.52	0.67
Organizational barriers^c^	Agency structure/systems, funding, attitudes, lack of vision	17	2.12	0.88
Personnel barriers^c^	Expertise, proprieties interest, availability, turnover	9	2.15	0.85
Perceived effectiveness^d^	Communication, decisions, coordination, service delivery	15	2.17	0.91
Perceived activity^d^	Rating of CP activity over 2 consecutive years	2	1.84	0.66
Role clarity^e^	Role perception matches that of participant	4	2.47	0.82
Operational understanding f	Knows CP mission, structure, operations	5	0.62	0.75
Communication mechanisms^g^	Use of newsletters, reports, meetings, etc.	7	0.45	0.68
Rules and procedures^h^	Operating principles, member orientation, mission, etc.	9	0.58	0.78
Previous CP experience^i^	Past experience of members in other partnerships	1	11	—

* Cronbach Alpha; CP: community partnership; HPE: health personnel education; PHC: primary health care;

† All sections scored on 7-point scales, higher ratings indicate a more ‘positive perception’, except;

a higher ratings indicate more contributions;

b higher ratings indicate more costs;

c Scored on 3-point scales, higher ratings indicate that barriers are less of a problem;

d Scored on 4-point scales, higher ratings indicate less effectiveness and less activity respectively;

e Scored on 5-point scales, higher ratings indicate more (higher level of) input (e.g. from advice only, to develop, recommend, or approve the CP ‘s budget, goals, comprehensive plan);

f Scored on 2-point scales, higher ratings indicate a more ‘positive perception’;

g categorical variable (YES/NO), overall probability (percentage) of YES answer ;

h categorical variable with three categories, overall probability (percentage) of YES answer;

i percentage of respondents reporting ‘YES’.

**Table 2.** Confirmatory items: 10 aspects of participants’ engagement and involvement^a^.

Item	Participants’ LSC^b^				P Value
	Low	Moderate	High	Excellent	
Past CP experience (% Yes)^c^	15.2	12.6	11.4	8.9	NS
Period since joining the CP (months)	18	21.6	20.5	24.2	0.036
CP meetings attended over last 12 months (%)	29.5	40.7	50.1	61.4	< 0.0001
Time spent on CP activity (hours per month)	18.38	20.66	29.44	26.6	NS
Since joining the CP, number of times participant:					
Recruited new members to the CP	5.73	8.53	7.17	12.1	0.027
Served as CP’s spokesperson	5.70	7	7.45	13.96	0.001
Served as CP’s representative to other groups	6.28	5.72	4.59	10.87	0.003
Implemented CP -sponsored educational/ culturalevents	6.22	9.18	7.74	14.27	0.006
Since joining the CP, how many:					
CP committees worked on	0.75	1.19	1.06	1.56	0.003
CP committee or team leadership positions held	0.22	0.31	0.31	0.61	< 0.0001

a All cells depict mean values (N= 668);

b LSC: Leadership Skills Category (Participants’ rating of leadership skills in their CPs);

c Those with past experience were 11% of the sample; CP: community partnership; NS: not significant.

**Table 3.** Participant ratings of partnership factors by perceptions of their leadership.

Factor	Participants’ rating of LSC* in their CPs				P Value
	Low	Moderate	High	Excellent	
A. Rated on 7-point scales**					
Management capabilities	3.66	4.19	4.78	5.42	< 0.0001
Community representation in the CP	3.56	3.92	4.84	5.17	< 0.0001
Staff-community member communication	3.48	3.43	4.42	5.15	< 0.0001
Community members communication	3.71	4.23	4.71	5.13	< 0.0001
Flow of information	3.41	3.83	4.46	5.12	< 0.0001
Participation Benefits	3.77	4.11	5.07	5.71	< 0.0001
Satisfaction with the CP	2.77	3.65	4.57	5.18	< 0.0001
Resource allocation satisfaction	2.28	2.82	3.32	4.65	< 0.0001
Staff expertise	4.03	4.27	4.93	5.53	< 0.0001
Community member expertise	3.81	4.05	4.57	4.95	< 0.0001
CP’s engagement in policy activities	2.80	3.06	4.27	4.38	< 0.0001
Partners’ effectiveness in policy activities	4.62	4.99	5.09	5.21	< 0.0001
CP’s engagement in HPE education	3.92	4.48	5.37	5.87	< 0.0001
Partners’ effectiveness in educational activities	3.83	4.50	5.39	5.90	< 0.0001
Sense of ownership	3.43	4.35	5.16	5.91	< 0.0001
Organisational commitment	3.67	4.22	4.95	5.66	< 0.0001
Interactions of the CP	3.20	4.03	4.70	5.27	< 0.0001
Decision-making	3.80	4.30	4.66	5.01	< 0.0001
Outcomes	3.23	3.80	4.53	5.30	< 0.0001
Contributions to the CP^a^	2.94	3.25	3.82	4.20	< 0.0001
Participation costs^b^	3.86	3.88	3.61	3.31	< 0.0001
**B. Rated on 3, 4 or 5-point scales**					
Organizational barriers^c^	1.65	1.89	2.09	2.27	< 0.0001
Personnel barriers^c^	1.69	1.93	2.09	2.30	< 0.0001
Perceived effectiveness^d^	2.65	2.49	2.23	2.01	< 0.0001
Perceived activity^d^	2.39	2.09	1.84	1.70	< 0.0001
Role clarity^e^	1.62	1.87	2.47	2.76	< 0.0001

CP: community partnership;

* LSC: Leadership Skills Category (participants’ rating of leadership skills in their CPs), all cells depict groups’ mean ratings;

** All sections scored on 7-point scales, higher ratings indicate a more ‘positive perception’

a higher ratings indicate more contributions;

b higher ratings indicate more costs;

c Scored on 3-point scales, higher ratings indicate that barriers are less of a problem;

d Scored on 4-point scales, higher ratings indicate less effectiveness and less activity respectively;

e Scored on 5-point scales, higher ratings indicate more (higher level of) input (e.g. from advice only, to develop, recommend, or approve the partnership’s budget, goals, comprehensive plan).

**Table 4.** Leadership Skills Score by participation costs to benefits ratio.

LSC	Comparison of difficulties with benefits of being a CP member				
	Many more difficulties than benefits	A few more difficulties than benefits	About the same amount of difficulties and benefits	A few more benefits than difficulties	Many more benefits than difficulties
Low to Moderate	37.4	18.3	22.9	14.5	6.9
High	20.7	19.2	16.6	24.4	19.2
Excellent	7.3	9.6	20.1	26.8	36.1

LSC: Leadership Skills Category, cells depict percentages of participants reporting ‘Yes’; P < 0.0001 *Leadership Skills and other 3 Partnership Factors.*

**Table 5.** Participant Leadership Skill Score for categories of selected CP Factors.

Factors	LSS†according to response to the item			P Value
	Yes	No	Don’t Know	
**Operational understanding**: knowledge of^a^				
How new members are chosen	0.79	0.64	—	< 0.0001
How committees/task forces are formed	0.78	0.64	—	< 0.0001
Organisational structure/staffing of the CP	0.76	0.61	—	< 0.0001
Clear understanding of mission of the CP	0.76	0.54	—	< 0.0001
One’s own role in the CP	0.75	0.53	—	< 0.0001
**Communication mechanisms**^a^				
Regularly published newsletters	0.78	0.65	—	< 0.0001
Written reports from staff	0.76	0.60	—	< 0.0001
Written reports from funded projects	0.78	0.66	—	< 0.0001
Verbal reports at CP and committee meetings	0.75	0.57	—	< 0.0001
Opportunities to talk with funded projects at meetings	0.77	0.66	—	< 0.0001
Talk with staff outside of meetings	0.76	0.63	—	< 0.0001
Talk with other CP members outside of meetings	0.77	0.67	—	< 0.0001
Talk with funded projects outside of meetings	0.78	0.70	—	< 0.0001
**Rules and procedures**: knowledge of whether the CP^b^				
Has written mission statement	0.75	0.62	0.56	< 0.0001
Has written by-laws/ operating principles	0.77	0.64	0.61	< 0.0001
Reviews its by-laws/ operating principles periodically	0.80	0.65	0.65	< 0.0001
Engages in strategic planning	0.74	0.59	0.58	< 0.0001
Has long-range plan beyond Kellogg funding	0.77	0.67	0.64	< 0.0001
Has written objectives	0.73	0.58	0.63	< 0.0001
Reviews its mission, goals and objectives periodically	0.76	0.61	0.64	< 0.0001
Has clear procedures for leader selection	0.77	0.62	0.67	< 0.0001
Provides orientation for new members	0.78	0.61	0.61	< 0.0001

CP: community partnership

† LSS: Leadership skill score (participants’ rating of leadership skills in their CPs), cells depict groups’ mean of LSS based on their response to the categories of each of the variables (rows), where higher leadership skill scores are associated with a ‘Yes’ response in comparison to ‘No’ or ‘Don’t Know’;

a response scales comprise ‘Yes/ No’ options;

b response scales comprise ‘Yes / No / Don’t Know’ options.
